# 

**Published:** 1874-01-01

**Authors:** 


THE EXAMINER *will be sent, after this Number, only to those who have renewed their Subscription for* 1874.

No. I. CHICAGO, JANUARY 1, 1874. Vol. XV.

### THE Medical Examiner.

### A *Semi-Monthly Journal of Medical Sciences.*

*TERMS.—Yearly Subscriptions, Three Dollars, in advance. Single Number Fifteen Cents. All Communications should be addressed to Publishers* MEDICAL EXAMINER, 65 *Randolph st., cor. State, Chicago.*

